# Laparoscopic partial versus radical nephrectomy for localized renal cell carcinoma over 4 cm

**DOI:** 10.1007/s00432-023-05487-3

**Published:** 2023-11-09

**Authors:** Zi-Jun Sun, Feng Liu, Hai-Bin Wei, Da-Hong Zhang

**Affiliations:** 1 Department of Urology, Zhejiang Provincial People’s Hospital, Qingdao University, Qingdao, Shandong China; 2 Urology and Nephrology Center, Department of Urology, Zhejiang Provincial People’s Hospital (Affiliated People’s Hospital), Hangzhou Medical College, No. 158 Shangtang Road, Gongshu District, Hangzhou, 310014 Zhejiang China

**Keywords:** Renal cell carcinoma, Partial nephrectomy, Radical nephrectomy, Renal function, Long-term prognosis

## Abstract

**Purpose:**

To compare the long-term clinical and oncologic outcomes of laparoscopic partial nephrectomy (LPN) and laparoscopic radical nephrectomy (LRN) in patients with renal cell carcinoma (RCC) > 4 cm.

**Methods:**

We retrospectively reviewed the records of all patients who underwent LPN or LRN in our department from January 2012 to December 2017. Of the 151 patients who met the study selection criteria, 54 received LPN, and 97 received LRN. After propensity-score matching, 51 matched pairs were further analyzed. Data on patients’ surgical data, complications, histologic data, renal function, and survival outcomes were collected and analyzed.

**Results:**

Compared with the LRN group, the LPN group had a longer operative time (135 min vs. 102.5 min, *p* = 0.001), larger intraoperative bleeding (150 ml vs. 50 ml, *p* < 0.001), and required longer stays in hospital (8 days vs. 6 days, *p* < 0.001); however, the level of ECT-GFR was superior at 3, 6, and 12 months (all *p* < 0.001). Similarly, a greater number of LRN patients developed CKD compared with LPN until postoperative 12 months (58.8% vs. 19.6%, *p* < 0.001). In patients with preoperative CKD, LPN may delay the progression of the CKD stage and even improve it when compared to LRN treatment. There were no significant differences between the two groups for OS, CSS, MFS, and PFS (*p* = 0.06, *p* = 0.30, *p* = 0.90, *p* = 0.31, respectively). The surgical method may not be a risk factor for long-term survival prognosis.

**Conclusion:**

LPN preserves renal function better than LRN and has the potential value of significantly reducing the risk of postoperative CKD, but the long-term survival prognosis of patients is comparable.

**Supplementary Information:**

The online version contains supplementary material available at 10.1007/s00432-023-05487-3.

## Introduction

Renal cell carcinoma (RCC), the most common type of urogenital cancer, accounts for approximately 2–3% of all malignant tumors and is more commonly seen in men than women (Motzer et al. 2022; Siegel et al. 2023). Based on cancer statistics report by the American Cancer Society, the incidence of RCC in both men and women in the United States has increased by about 1% per year since the mid-twentieth century (Siegel et al. 2023). Despite a steady increase in incidence, the mortality rate for RCC was reduced by about 2% per year from 2016 to 2020, which may be related to the early diagnosis and the increased nephrectomy rate (Medina-Rico et al. 2018; Motzer et al. 2022; Siegel et al. 2023).

For localized RCC, surgical treatment is preferred. With the application and development of laparoscopic techniques, laparoscopic partial nephrectomy (LPN) is recommended for patients with T1a (≤ 4 cm) tumors in the American Urological Association (AUA) guideline (Liss et al. 2014; Campbell et al. 2021). For larger renal tumors (> 4 cm), laparoscopic radical nephrectomy (LRN) is still the standard treatment (Rini et al. 2009; Lee et al. 2014), but in selected cases, the long-term efficacy of LPN is similar to that of LRN, and LPN has a better effect on the preservation of renal function (Ching et al. 2013; Tuderti et al. 2022, 2023).

However, the “optimal” surgical treatment for renal tumors > 4 cm is still disputed, and not all studies indicate that LPN is preferable to LRN. As exhibited in some studies, compared with the LRN group, LPN had a higher risk of complications such as bleeding (Mir et al. 2017). In terms of prognosis, a prospective randomized-controlled study, EORTC 30904, found that LPN was not superior to LRN in patients with overall survival (OS) (Scosyrev et al. 2014). Based on the regression analysis of competing-risks data, after mortality rates from other causes are adjusted, there is no statistically significant correlation between nephrectomy type and cancer-specific mortality rate (Meskawi et al. 2014). Based on these debates, there have been no definitive conclusions made about the role of LPN for RCC > 4 cm.

Thus, this study compared the long-term clinical and oncologic outcomes between LPN and LRN, so as to provide references for the clinical treatment of RCC.

## Materials and methods

### Study population

This retrospective study collected information on patients with RCC who had their initial surgery, either LPN or LRN in Zhejiang Provincial People’s Hospital from January 2012 to December 2017. The study protocol conforms to the ethical guidelines of the 2013 Declaration of Helsinki. The informed consent processes had been approved by the ethics committees in Zhejiang Provincial People’s Hospital (Approval No. 2021QT082) before the study started.

The inclusion criteria were: (1) meeting the 2023 NCCN guideline, radiological, or histological diagnostic criteria of RCC before surgery; (2) normal renal function on the healthy side, with a solitary tumor on the affected side; (3) undergoing LRN or LPN with localized renal masses measuring > 4 cm. (4) no obvious abdominal mass or another advanced renal carcinoma.

The exclusion criteria were: (1) presenting contralateral solitary, atrophic, or congenital absent kidney; (2) history of any other kind of tumors or recrudescent tumors of the kidney; (3) distant metastasis; (4) severe disease of the heart, lungs, kidneys, brain, blood, or other vital organs before operation; (5) complicated with systemic disease; (6) performed open surgery, interventional surgery, or non-surgical treatment; (7) performed other surgeries at the same time; and (8) incomplete clinical data.

### Data collection and follow-up

The baseline date was defined as the date RCC patients underwent their initial surgery. Patients’ demographics, clinical characteristics, tumor localization, tumor size, tumor stage, preoperative American Society of Anesthesiologists (ASA) score, and test results of biochemistry, and ultrasonography were collected. Meanwhile, information regarding operation time, ischemia time, postoperative pathology (Sup. 1), positive surgical margin, intraoperative complications, postoperative complication score, and hospital stay was also collected. The postoperative complication score was evaluated on the basis of the Clavien–Dindo Classification of Surgical Complications (Dindo et al. 2004).

Kidney function evaluation included SCr and emission computed tomography for glomerular filtration rate measurement (ECT-GFR, ml/min) preoperatively and before discharge and at postoperative 3 months, 6 months, and 12 months (Sup. 2). The follow-up date was defined as the specific time of follow-up every 3 months after the patient was discharged from the hospital. The patients were followed every 3 months within months 6, semi-annual for up to 3 years, annually thereafter for survival and disease status, including sites of first recurrence and post-recurrence survival. Endpoint events were defined as recurrence, metastasis, and death during follow-up. According to the collected data, the occurrence of end-point events in RCC patients treated with LPN and LRN during the follow-up period was statistically analyzed, as were the related influencing factors.

### Surgical technique

Surgical procedures for all patients were performed the standard transperitoneal approach. Endotracheal intubation and general anesthesia were carried out on patients under a lateral position with the normal side down to fully expose the surgical field of the affected side. With a Trocar approach, a laparoscope was inserted to make pneumoperitoneum to expand the surgical space. The Gerota fascia was opened with an ultrasonic scalpel to completely dissociate the kidney on the affected side from bottom to top, so as to expose renal tumors. For LPN, the renal artery was blocked with a bulldog clamp and the tumor was resected with a margin of 0.5–1.0 cm of healthy renal parenchyma. And for LRN, after the renal artery on the affected side was blocked, the kidney, the surrounding tissue, and the fascia were completely excised, and then, the ruptured blood vessels were sutured. The pneumoperitoneum was closed after there was no abnormality, the Trocar was withdrawn, and the drainage tube was placed beside the kidney. Finally, the puncture was closed to finish the surgery.

### The outcome assessment

The primary outcomes were the effects of different surgical methods on renal function and the incidence of chronic kidney disease (CKD), which was defined as GFR < 60 ml/min over 3 months. Meanwhile, the long-term survival prognosis and influencing factors of the patients were also observed.

The secondary outcomes included the following: (1) operative time, intraoperative bleeding, and postoperative hospital stay, (2) incidence rate of postoperative complications, and (3) postoperative pathological features.

### Statistical analysis

Continuous variables are expressed as mean ± SD or median (IQR), while categorical variables are presented as *n* (%). Qualitative and quantitative differences between the two groups were analyzed by the Chi-square or Fisher’s exact test for categorical variables, and the Student’s *t* test or Mann–Whitney *U* test for continuous variables, as appropriate.

To minimize the biasing effects of confounders, a 1:1 caliper width of 0.2 for the propensity-score matching (PSM) analysis was performed on the following variables: tumor size, cTNM stage, preoperative Hb, and SCr. Pairs of patients were selected using the “nearest-neighbor” matching method. The log-rank test was used to compare the Kaplan–Meier estimates for overall survival (OS), cancer-specific survival (CSS), metastasis-free survival (MFS), and progression-free survival (PFS). OS was calculated from the date of surgery to the date of death for any reason; CSS was calculated from the date of surgery to the date of death caused by RCC; MFS was calculated from the date of surgery to the date of the first-time tumor metastasis, or the death of any cause; PFS was calculated from the date of surgery to the date of disease progression, or death. For the univariate and multivariate analyses, the Cox proportional hazard regression model was applied to estimate the prognostic risk factors of patients.

All statistical tests were two-sided. Statistical significance was set at *p* < 0.05. Statistical analysis was performed with IBM SPSS Statistics Version 25.0 (IBM Corp., Armonk, NK, USA), GraphPad Prism Version 8.0.0 (http://www.graphpad.com), and R version 4.0.4 (http://www.r-project.org).

## Results

### Patients’ enrollment and baseline characteristics

**Fig. 1:**
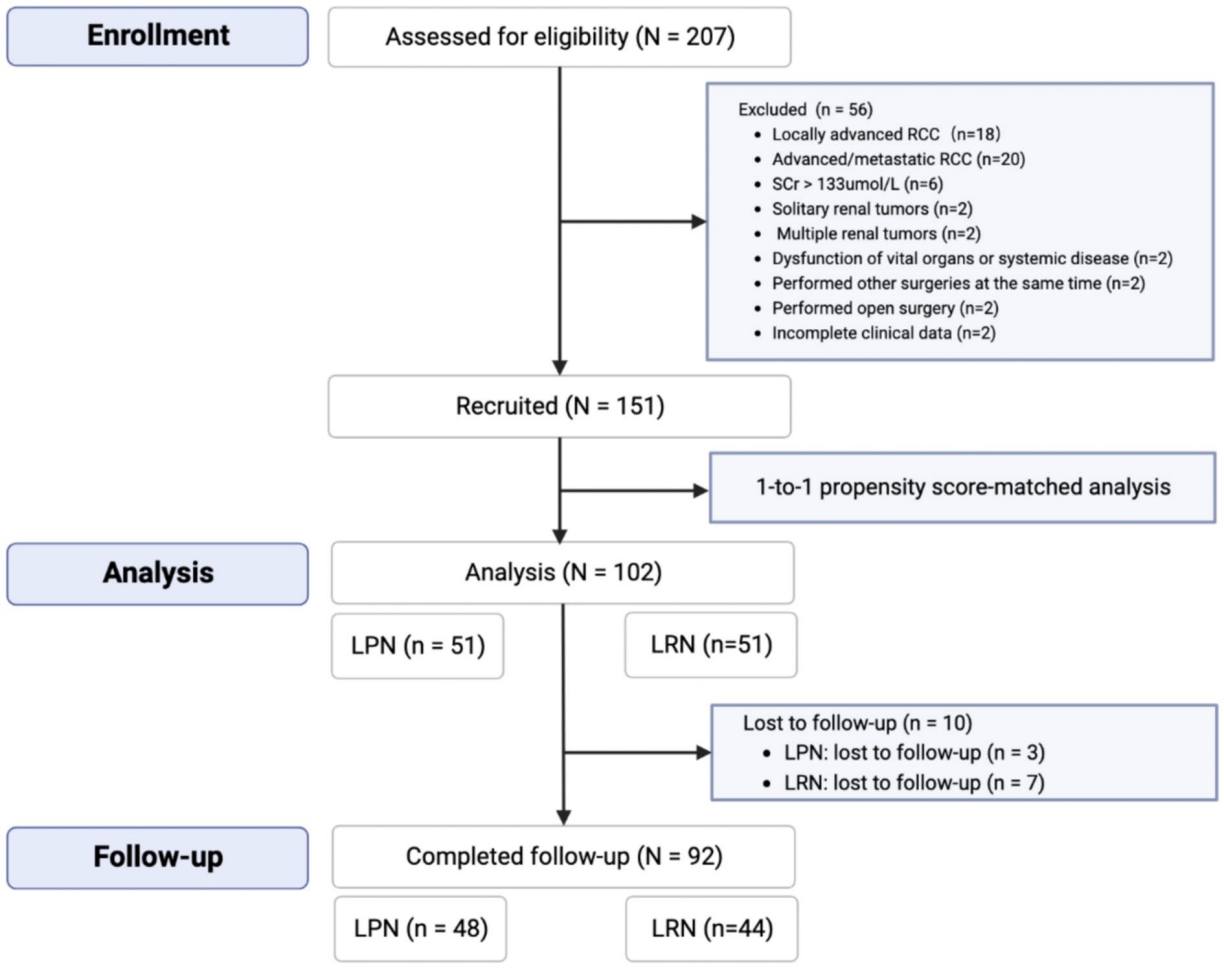
Flowchart of patient characteristics

During the study period, 207 patients with RCC > 4 cm were screened retrospectively for eligibility at Zhejiang Provincial People’s Hospital. After excluding 56 patients for various reasons, 151 patients were enrolled. In this study, the PSM analysis was further used to match the baseline of the two groups. After matching, a total of 102 people were included in the final study, with 51 people in each group. Of the remaining 102 patients, 92 completed the follow-up with outcome data collected (Fig. 1).

The patients’ baseline characteristics before and after propensity-score matching are shown in Table 1. Before propensity-score matching, more patients in the LRN group had a significantly larger tumor size (6 cm vs. 5 cm) and higher cTNM stage than the LPN group. In addition, some test results of biochemistry like Hb (*p* = 0.036) and SCr (*p* = 0.008) before surgery also had differences between the two groups. After matching, all items were seen to have no significant statistical difference when comparing both groups (*p* > 0.05).

**Table 1:** Clinical characteristic of patients with RCC over 4 cm

Variable	All patients (*n* = 151)			Propensity-score matched patients (*n* = 102)		
	LPN (*n* = 54)	LRN (*n* = 97)	*p* value	LPN (*n* = 51)	LRN (*n* = 51)	*p* value
Age (years)	56.41 ± 14.52	58.44 ± 12.76	0.37	56.10 ± 14.47	58.24 ± 13.05	0.44
Gender, *n* (%)			0.49			0.53
Male	35 (64.8%)	56 (57.7%)		32 (62.7%)	36 (70.6%)	
Female	19 (35.2%)	41 (42.3%)		19 (37.3%)	15 (29.4%)	
BMI (kg/m^2^)	23.51 (21.86, 25.91)	23.35 (20.96, 25.75)	0.57	23.77 ± 2.90	23.98 ± 2.92	0.72
BMI, *n* (%)			0.79			> 0.99
< 18.5	3 (5.6%)	7 (7.2%)		3 (5.9%)	3 (5.9%)	
18.5–24.9	32 (59.3%)	59 (60.8%)		31 (60.8%)	31 (60.8%)	
25.0–29.9	18 (33.3%)	27 (27.8%)		16 (31.4%)	16 (31.4%)	
≥ 30.0	1 (1.8%)	4 (4.2%)		1 (1.9%)	1 (1.9%)	
Smoking, *n* (%)	13 (24.1%)	28 (28.9%)	0.57	12 (23.6%)	15 (29.4%)	0.65
Drinking, *n* (%)	8 (14.8%)	19 (19.6%)	0.51	8 (15.7%)	8 (15.7%)	> 0.99
Hypertension, *n* (%)	17 (31.5%)	41 (42.3%)	0.22	15 (29.4%)	21 (41.2%)	0.30
CHD, *n* (%)	1 (1.9%)	3 (3.1%)	> 0.99	0 (0%)	2 (3.9%)	0.50
Diabetes, *n* (%)	4 (7.4%)	10 (10.3%)	0.77	4 (7.8%)	5 (9.8%)	> 0.99
Laterality, *n* (%)			> 0.99			0.84
Right	29 (53.7%)	52 (53.6%)		27 (52.9%)	25 (49%)	
Left	25 (46.3%)	45 (46.4%)		24 (47.1%)	26 (51%)	
Tumor size (cm)	5.00 (4.50, 6.00)	6.00 (5.00, 7.50)	0.001^a^	5.00 (4.50, 6.00)	5.50 (4.50, 6.50)	0.56
cTNM, *n* (%)			0.001^a^			0.74
I	50 (92.6%)	68 (70.1%)		47 (92.2%)	45 (88.2%)	
II	4 (7.4%)	29 (29.9%)		4 (7.8%)	6 (11.8%)	
Hb (g/l)	143.00 (131.00, 154.30)	137.00 (122.50, 149.50)	0.04^a^	141.70 ± 13.01	140.60 ± 16.47	0.72
Ca (mmol/l)	2.31 (2.24, 2.39)	2.29 (2.24, 2.36)	0.38	2.31 ± 0.10	2.31 ± 0.12	0.83
SCr (umol/l)	89.28 ± 17.97	81.74 ± 15.83	0.01^a^	87.18 ± 16.13	86.13 ± 16.39	0.74
LDH (U/l)	169.00 (149.00, 196.00)	170.50 (154.30, 188.30)	0.88	171.00 (152.00, 188.00)	172.00 (151.00, 197.00)	0.54
ECT-GFR (ml/min)	73.17 ± 19.27	76.44 ± 20.44	0.34	74.14 ± 19.18^a^	74.54 ± 20.24	0.92
ASA, *n* (%)			0.16			0.09
1	9 (16.7%)	19 (19.6%)		8 (15.7%)	11 (21.6%)	
2	43 (79.6%)	66 (68.0%)		42 (82.4%)	34 (66.6%)	
3	2 (3.7%)	12 (12.4%)		1 (1.9%)	6 (11.8%)	
4	0 (0%)	0 (0%)		0 (0%)	0 (0%)	

Continuous variables were expressed as mean ± SD or median (IQR), while categorical variables were presented as *n* (%)

BMI, body Mass Inde; CHD, coronary heart disease; before surgery, all patients with a history of CHD were well controlled and without surgical contraindications; cTNM, clinical TNM classification; Hb, hemoglobin; Ca, serum calcium; SCr, serum creatinine; LDH, lactic dehydrogenase; ECT-GFR, emission computed tomography for glomerular filtration rate measurement; ASA, American Society of Anesthesiologists

^a^*p* < 0.05 was considered statistically significant

### The comparison of surgery-related indicators

Patients treated with LPN had longer operation time (135 min vs. 102.5 min, *p* = 0.001), hospital stay (8 days vs. 6 days, *p* < 0.001), and larger intraoperative bleeding (150 ml vs. 50 ml, *p* < 0.001) in comparison to patients receiving LRN. Other items like a positive surgical margin, intraoperative or postoperative complications, and even postoperative histology were seen to have no significant statistical difference. More details are shown in Table 2 and Tables S1–S2.

**Table 2:** The comparison of surgery-related indicators

Variable	LPN (*n* = 51)	LRN (*n* = 51)	*p* value
Operation time (min)	135.00 (100.00, 160.00)	102.50 (85.00, 125.00)	0.001^a^
Intraoperative bleeding (ml)	150.00 (100.00, 300.00)	50.00 (50.00, 150.00)	< 0.001^a^
Blood transfusion, *n* (%)	1 (1.9%)	2 (3.9%)	0.56
Intraoperative complication, *n* (%)	4 (7.8%)	1 (1.9%)	0.17
Postoperative complication, *n* (%)	22 (43.1%)	15 (29.4%)	0.15
Clavien–Dindo I–II	19 (37.2%)	14 (27.5%)	0.50
Clavien–Dindo ≥ III	3 (5.9%)	1 (1.9%)	0.72
Positive surgical margin, *n* (%)	0 (0%)	0 (0%)	> 0.99
Hospital stay (days)	8.00 (6.00, 10.00)	6.00 (5.00, 7.00)	< 0.001^a^

Continuous variables were expressed as median (IQR), while categorical variables were presented as *n* (%)

^a^*p* < 0.05 was considered statistically significant

### Efficacy of different surgery on postoperative renal function

**Fig. 2:**
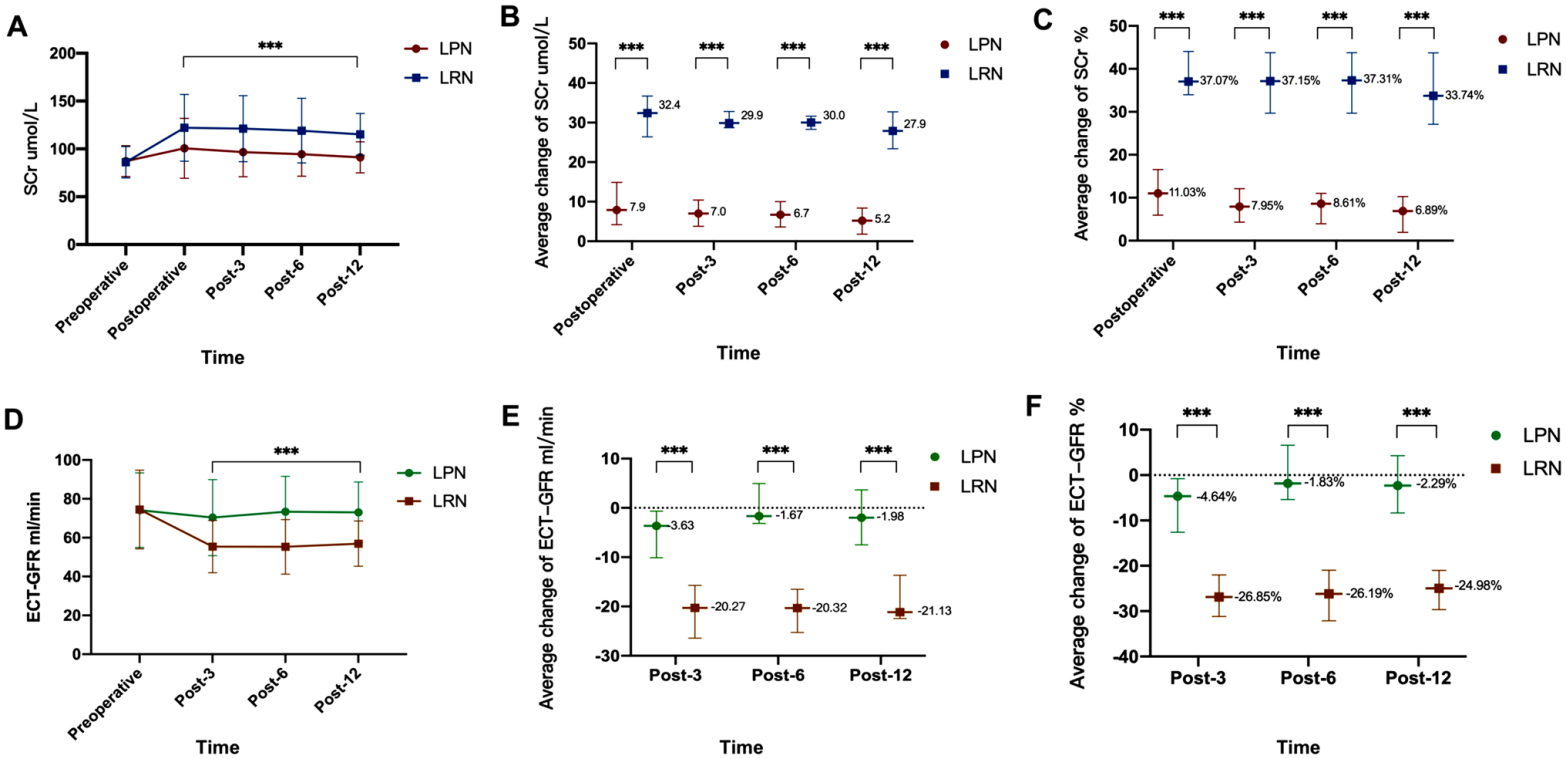
Comparison of renal function before and after operation between the two groups. **A** Mean SCr value at baseline and follow-up in patients in the LPN and LRN group. The differences in SCr value in these 2 groups at each time point were compared using Student’s *t* tests. **B** Comparison of postoperative SCr values changes (95% confidence interval) between the LPN and LRN groups. **C** Comparison of the percentage change (95% confidence interval) of SCr after surgery between LPN and LRN groups. **D** Mean ECT-GFR value at baseline and follow-up in patients in the LPN and LRN group. The differences in ECT-GFR value in these 2 groups at each time point were compared using Student’s *t* tests. **E** Comparison of postoperative ECT-GFR values changes (95% confidence interval) between the LPN and LRN groups. **F** Comparison of the percentage change (95% confidence interval) of ECT-GFR after surgery between LPN and LRN groups. The * means *p* < 0.05

The renal function tests were remarkably improved in the LPN group, as shown in Fig. 2. There was no meaningful statistical difference in the average preoperative SCr levels between the LRN and LPN groups. However, before discharge and at the following 3rd, 6th, and 12th months, the mean postoperative SCr values in the LPN group were significantly lower than those in the LRN group (all *p* < 0.001) (Fig. 2a). In the LRN group, we saw a significant increase from the baseline in the mean SCr at each subsequent follow-up (all *p* < 0.001). Overall, the mean difference in SCr levels between the two groups with a 95% confidence interval is shown in Fig. 2b, c. The net increases of SCr in the LRN group were significantly higher compared with the LPN group after the operation (all *p* < 0.001). In contrast to LRN, the level of ECT-GFR was superior in LPN at the postoperative time of 3 months, 6 months, and 12 months (all *p* < 0.001) (Fig. 2d–f).

**Fig. 3:**
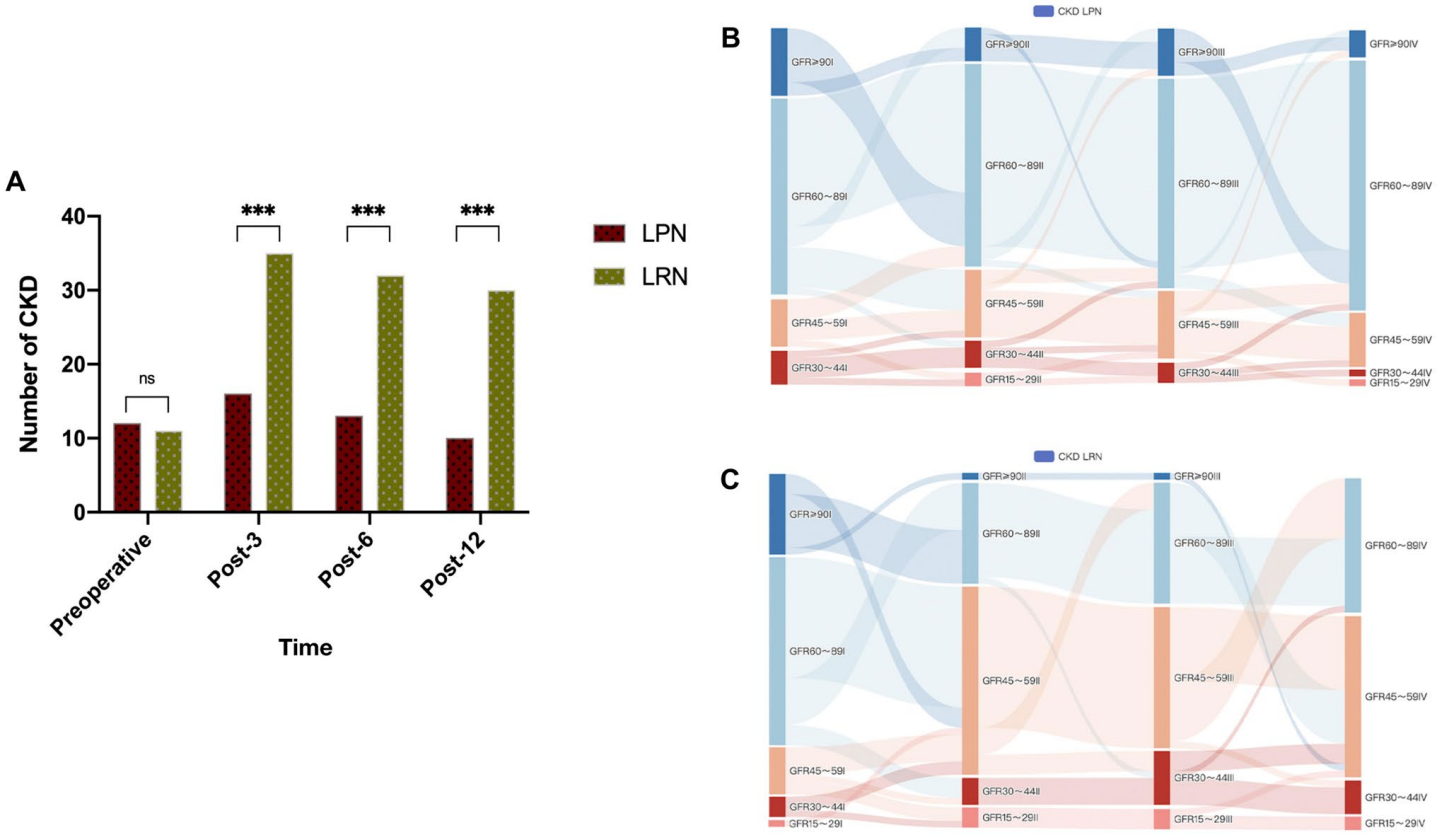
Comparison of the occurrence of CKD before and after operation between the two groups. **A** Comparison of the number of CKD (GFR < 60 ml/min) for patients after LPN or LRN for RCC > 4 cm. The * means *p* < 0.05. Sankey diagrams for the change of GFR from baseline to postoperative 12 months in the 2 groups. Sankey diagrams were used to show the major transfers or flows of patients. The colors of the columns represent patients with different GFR levels, with blue representing GFR ≥ 90 ml/min, green representing GFR 60–89 ml/min, orange representing GFR 45–59 ml/min, red representing GFR 30–44 ml/min, and pink representing GFR 15–29 ml/min. The length of the column represents the proportion of patients. The thicker the line, the greater the number of patients involved. I indicate preoperative; II indicates 3 months after operation; III indicates 6 months after surgery; IV indicates 12 months after surgery. **B** The LPN group (*n* = 51). **C** The LRN group (*n* = 51)

In addition, we also assessed the rate of chronic kidney disease (CKD: GFR < 60 ml/min) and confirmed the results of CKD rate in the LPN group to be significantly lower on the following 3rd, 6th, and 12th (all *p* < 0.001) months (Fig. 3a, Table S3). We plotted the Sankey diagram to represent the change of GFR from baseline to the following 12 months, which showed remarkably more favorable results in the LPN group (Fig. 3b, c). Additionally, as shown in Fig. 3, patients with preoperative GFR ≥ 60 ml/min in the LPN group rarely experienced renal function deterioration or CKD, even remarkably improving in the following 12 months after the operation. Most patients with preoperative GFR ≥ 60 ml/min in the LRN group developed CKD and did not improve at the last follow-up in patients with preoperative CKD.

### Comparison of survival between the two groups

**Fig.4:**
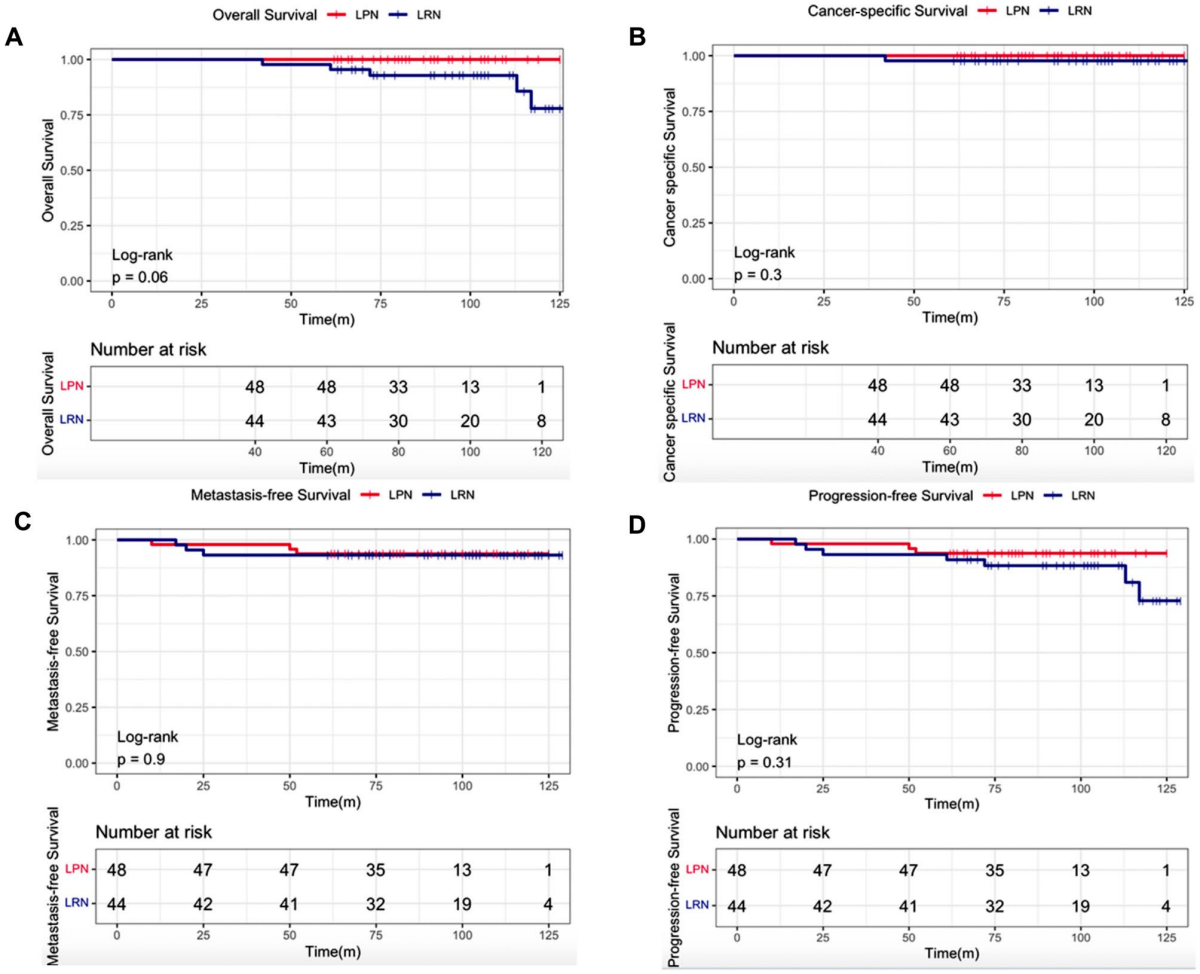
Kaplan–Meier analysis of overall survival (**A**, *p* = 0.06), cancer-specific (**B**, *p* = 0.30), metastasis-free (**C**, *p* = 0.90), and progression-free survival (**D**, *p* = 0.31) for patients after LPN or LRN for RCC > 4 cm

The follow-up deadline was set for February 25, 2023. Ten patients (3 cases in the LPN group and 7 cases in the LRN group) were lost to follow-up, and the loss rate was 9.8%. Finally, 92 patients completed long-term follow-ups. The median duration for follow-up completion was 88 months for the LPN group and 98 months for the LRN cohort. While completing the follow-ups for the study, 5 (11.4%) patients in the LRN cohort and no patients in the LPN group died (*p* = 0.022). In the LRN group, only 1 patient experienced cancer-related deaths, and the other 4 patients died because of non-cancer-related causes (*p* = 0.360). Both groups had 3 patients who experienced local recurrence or developed distant metastatic diseases, respectively (*p* > 0.99). The patients with metastases underwent surgical resection or targeted therapy, except for one who refused treatment. The OS, CSS, MFS, and PFS curves are depicted in Fig. 4.

### Factors associated with survival

Univariate analyses revealed several baseline variables associated with the OS, including age, a history of coronary heart disease (CHD), preoperative hemoglobin (Hb), preoperative lactic dehydrogenase (LDH), ASA score, and postoperative histology (all *p* < 0.05). Multivariate analysis showed that the following 5 baseline variables independently associated with the lower OS: higher age [hazard ratio (HR) 477.6; 95% CI 276.3–825.4; *p* < 0.001], a history of CHD (HR 1.831e−57; 95% CI 3.581e−60–9.362e−55; *p* < 0.001), lower preoperative Hb (HR 0.460; 95% CI 0.360–0.590; *p* < 0.001), higher ASA score (HR 1.224e+26; 95% CI 2.485e+23–6.033e+28; *p* < 0.001), and postoperative histology (HR 2.851e+25; 95% CI 6.885e+23–1.180e+27; *p* < 0.001) (Table 3). However, the surgical method was not found to be associated with survival. When examining all patients, univariate analysis showed that drinking (HR, 5.476; 95% CI, 1.105–27.139; *p* = 0.037) and ASA score (HR, 4.998; 95% CI, 1.575–15.792; *p* = 0.006) were the prognostic factors of MFS and PFS, respectively. No variables were independently associated with MFS or PFS in multivariate analysis, as well as CSS (Tables S4–S6).

**Table 3:** Univariate and multivariable analyses of prognostic risk factors for OS

Variable	OS			
	Univariate		Multivariate	
	HR (95% CI)	*p* value	HR (95% CI)	*p* value
Age (years)	1.223 (1.047–1.428)	0.01^b^	4.776e+02 (2.763e+02–8.254e+02)	< 0.001^b^
Gender (male vs. female)	0.557 (0.062–5.046)	0.60		
BMI	0.902 (0.657–1.237)	0.52		
Surgical method (LRN vs. LPN)	57.679 (0.022–1.507e+05)	0.31		
Smoking (no vs. yes)	0.736 (0.082–6.595)	0.78		
Drinking (no vs. yes)	0.036 (0.000–679.222)	0.51		
Hypertension (no vs. yes)	2.237 (0.372–13.467)	0.38		
Diabetes (no vs. yes)	0.043 (0.000–4.811e+04)	0.66		
CHD (no vs. yes)	20.874 (1.888–230.783)	0.01^b^	1.831e−57 (3.581e−60–9.362e−55)	< 0.001^b^
Laterality (Left vs. Right)	0.693 (0.115–4.160)	0.69		
Tumor size	1.630 (0.819–3.246)	0.16		
pT stage (pT1b vs. pT2a)	0.041 (0.000–5.709e+03)	0.60		
Hb	0.939 (0.882–0.999)	0.046^b^	0.460 (0.360–0.590)	< 0.001^b^
Ca	0.000 (0.000–3.168)	0.09		
SCr	1.027 (0.979–1.076)	0.27		
LDH	1.015 (1.003–1.028)	0.01^b^	0.99 (0.92–1.07)	0.80
ECT-GFR	0.972 (0.933–1.012)	0.17		
Preoperative CKD (no vs. yes)	4.684 (0.777–28.228)	0.09		
ASA score (1 vs. 2 vs. 3)	13.399 (2.369–75.787)	0.003^b^	1.224e+26 (2.485e+23–6.033e+28)	< 0.001^b^
Operation time	0.987 (0.958–1.016)	0.36		
Intraoperative bleeding	0.997 (0.989–1.005)	0.47		
Hospital stay	0.856 (0.580–1.262)	0.43		
Postoperative complication (no vs. yes)	1.834 (0.289–11.634)	0.52		
Postoperative SCr	1.007 (0.981–1.034)	0.61		
Postoperative ECT-GFR	0.962 (0.912–1.016)	0.16		
Postoperative CKD (no vs. yes)	3.525 (0.391–31.745)	0.26		
Postoperative histology^a^	3.000 (1.156–7.785)	0.02^b^	2.851e+25 (6.885e+23–1.180e+27)	< 0.001^b^
Local recurrence (no vs. yes)	3.755 (0.418–33.718)	0.24		
Distant metastatic diseases (no vs. yes)	0.049 (0.000–1.235e+19)	0.90		

OS, overall survival; HR, hazard ratio; BMI, body Mass Index; CHD, coronary heart disease; before surgery, all patients with a history of CHD were well controlled and without surgical contraindications; Hb, hemoglobin; Ca, serum calcium; SCr, serum creatinine; LDH, lactic dehydrogenase; ECT-GFR, emission computed tomography for glomerular filtration rate measurement; CKD, chronic kidney disease; ASA, American Society of Anesthesiologists; 95% CI: 95% confidence interval

^a^Represents postoperative histology (clear cell renal cell carcinoma vs. papillary renal cell carcinoma vs. other types)

^b^*p* < 0.05 was considered statistically significant

## Discussion

Our current study shows that in patients with RCC > 4 cm, LPN has better renal function and a comparable oncology outcome than the LRN group. Additionally, the surgical method in our study may not be a risk factor for long-term survival.

In this study, after adjusting for differences in baseline characteristics through PSM analysis, 102 patients with RCC > 4 cm were included, of whom 51 received LRN treatment and the remaining 51 received LPN treatment. To our knowledge, although we are not the first study to report on the surgical treatment of patients with RCC > 4 cm, we have made some additional findings and supplements in terms of changes in postoperative long-term renal function, particularly CKD, and the influencing factors of patient survival prognosis. Furthermore, the study's follow-up time was adequate, with an average follow-up time of 7.5 years.

The basic principles of nephron-sparing surgery (NSS) for RCC are complete excision of the lesion with negative margins while preserving as many viable renal parenchymas as possible (Deklaj et al. 2010; Alyami and Rendon 2013). It emerged that the LPN cohort was superior in both levels of SCr and its changes, as well as ECT-GFR, as shown by the previous studies (Kaushik et al. 2013; Larcher et al. 2016; Cai et al. 2018; Yang et al. 2020). The operation requires temporary blockade of the renal artery during surgery, which can cause ischemia–reperfusion injury in the kidney, and with the extension of renal warm ischemia time (WIT), it will cause irreversible damage to the remaining renal function. To preserve remaining renal function and reduce blood loss, preoperative superselective transarterial embolization (STE) was first described by Gallucci et al. as an option to perform LPN without hilar clamping (Gallucci et al. 2007). Our findings indicated that patients who underwent LPN had negative surgical margins, with a median warm ischemia time (WIT) of 20 min and a maximum WIT of 30 min, and 80.4% (41/51) of them had a WIT time < 25 min. This suggested that as surgical technology advances and surgeons gain experience, WIT shortens, ischemia injury to residual kidneys is minimized, and LPN preserves more postoperative nephrons in patients and plays an even greater protective role in renal function (Gallucci et al. 2007; Simone et al. 2009; Rajan et al. 2016; Mehra et al. 2019; Takeda et al. 2020).

The protection of renal function after LPN depends on the preoperative GFR, WIT, and the amount of renal parenchyma preserved after the operation (Thompson et al. 2012; Rogers et al. 2013). These determinants in turn influence the development of CKD, which is a recognized risk factor for anemia, hypertension, malnutrition, and neurological diseases (Huang et al. 2006). It is associated with poorer quality of life in patients, a higher risk of hospitalization, the occurrence of cardiovascular events, and death. Notably, data on patients further demonstrated fewer CKD after LPN procedures, which showed agreement with the available literature (Kaushik et al. 2013; Larcher et al. 2016). However, unlike previous studies, we also included patients with preoperative CKD and stratified them according to their stage. After visualizing the data with the Sankey diagram, it was found that the patients with GFR < 60 ml/min in both groups were similar before surgery (12 vs. 11). Until the 12th month follow-up, only 1 patient in the LPN group developed to CKD stage IV, while 6 patients showed improvement in ECT-GFR. In the LRN group, 2 patients developed stage IV, and 1 patient showed improvement in ECT-GFR. Though there was no statistical difference in postoperative progression (*p* = 0.590) and improvement (*p* = 0.069) between the two groups of preoperative CKD patients, LPN may have protected postoperative renal function. Combining the results of others’(Britton et al. 2022), we believe that LRN was more likely to result in a decline in GFR and CKD stage progression compared to LPN for patients with preoperative GFR < 60 ml/min. Thus, it is reasonable to persuade that LPN can prevent or delay renal, cardiovascular, and other debilitating systemic impairments by providing the benefit of preserving renal function.

Another contentious matter for LPN and LRN is the oncological outcomes, which play a major role in the improvement of life expectancy. Kopp et al. (2014) revealed that there was no difference in survival for LRN versus LPN by Kaplan–Meier analysis for OS, CSS, or PFS. Similarly, our research found that there was no statistical difference, although the LPN group offered a superior OS (*p* = 0.060) than patients undergoing LRN. However, compared to previous reports, we did not find that the improvement of renal function after LPN improves the patients’ survival, like CSS, MFS, or PFS (Scosyrev et al. 2014; Tobert et al. 2014; Jang et al. 2016). Interestingly, we found that low preoperative Hb and high preoperative LDH were risk factors for OS. Similarly, preoperative low Hb has been considered a risk factor for OS in patients with RCC, especially those with renal venous cancer embolus (Abel et al. 2017; Peng et al. 2018). Hb, along with serum calcium ion and alkaline phosphatase (ALP) levels, is also considered to be a risk factor for advanced bone metastasis in patients with RCC (Hu et al. 2020; Kaul et al. 2021). In addition, previous studies have shown that higher serum LDH level is a poor prognostic factor for PFS (HR = 1.74, 95% CI 1.48–2.04, *p* < 0.001) (Shen et al. 2016). Besides, LDH has been shown to be an independent prognostic marker for patients with metastatic RCC (Shen et al. 2016). However, this study indicated that higher preoperative LDH levels were linked with OS in univariate analysis but not in multivariate analysis. LDH has been previously confirmed to participate in glycolysis of tumor cell metabolism, provide energy for tumor cells, directly inhibit apoptosis, avoid necrosis of tumor cells in a hypoxia environment, and thus promote tumor growth. Hence, it can be postulated that the observed disparity may be attributed to the influence of patients' nutritional status and other diseases on LDH levels. Therefore, we boldly speculate that the use of LPN may not depend on the clinical stage or the size of the tumor, but on the individual patient characteristics and technical ability of the physician to remove the tumor (Mir et al. 2017).

In our study, postoperative histology was also an independent factor of OS. Its impact on survival is still controversial. A large study from China examined the effect of postoperative pathologic types on the prognosis of patients with RCC, using the SEER database as a reference (Shao et al. 2021). The results showed that, among the 1346 patients included, clear cell renal cell carcinoma in the Huaxi database offered a superior OS than papillary renal cell carcinoma (Shao et al. 2021). However, the opposite is true in the SEER database: papillary renal cell carcinoma had a better OS than clear cell renal cell carcinoma. Our results are similar to those found in the SEER database (Fig. S1). Due to the limited sample size, some biases may have been introduced in our study. The effects of postoperative histology on survival still need to be studied with a large sample size.

In addition, the study also focused on intraoperative indicators and postoperative complications. We tend to use the transperitoneal approach to provide a larger space in the surgery (Takagi et al. 2021). The median operation time for the LRN cohort was significantly short compared with the LPN group (102.5 min vs. 135 min, *p* = 0.001). It is logically concerning that the technical procedures when performing LPN are more challenging than LRN for large renal masses, which do not need the suture of the kidney (Yang et al. 2020). Similar to previous reports (Deklaj et al. 2010; Rinott Mizrahi et al. 2018), we also found greater intraoperative bleeding in the LPN group (150 ml vs. 50 ml, *p* < 0.001). Importantly, there was no increase in the incidence of complications both intraoperative and postoperative in patients who had LPN when compared with patients who received LRN. Obviously, for large tumors, LPN has limitations such as reduced operating space, increased operating difficulty, and larger surgical wound surface, which increases the intraoperative suture time, intraoperative bleeding, and even hospital stay.

Our study has some limitations. First, as a result of matching the majority of patients in the original sample using PSM analysis, the sample size was insufficient to fully utilize the collected data. Second, the baseline did not include indicators like the R.E.N.A.L. score to quantify tumor location and other anatomic features and complexity due to retrospective study data limitations. Furthermore, certain patients were lost to follow-up during the procedure of long-term follow-up. Due to the insufficient number of patients with RCC > 7 cm, further analysis is also not feasible. Thus, it is imperative to conduct prospective, controlled, and randomized trials that incorporate a more extensive database and have an extended follow-up period in order to investigate long-term clinical and survival outcomes in greater depth.

In conclusion, we validated that both LPN and LRN were safe and effective for patients with localized RCC > 4 cm. The surgical methods did not affect the survival prognosis of patients. LPN effectively protects renal function and has the potential to drastically reduce the risk of postoperative CKD. However, the risk of intraoperative and postoperative bleeding, as well as a longer hospital stay for LPN, must still be considered. In this study, we recommend LPN as a treatment option for patients with localized RCC > 4 cm, but the choice of LPN should be based on individual patient characteristics, surgeon expertise, and technological feasibility.

### Supplementary Information

Below is the link to the electronic supplementary material.

**Supplementary file 1. Fig. S1**: Kaplan–Meier curve of the influence of postoperative pathological types on OS. The red line represents clear cell renal cell carcinoma (ccRCC), the dark blue line represents papillary renal cell carcinoma (pRCC), and the light blue line represents other pathologic types. (TIFF 53 kb)

Supplementary file 2 (DOCX 67 kb)

## Data Availability

The datasets generated during and analyzed during the current study are available from the corresponding author on reasonable request.

## Abbreviations

LPN: Laparoscopic partial nephrectomy
LRN: Laparoscopic radical nephrectomy
RCC: Renal cell carcinoma
OS: Overall survival
SCr: Serum creatinine
ASA: American Society of Anesthesiologists
ECT-GFR: Emission computed tomography for glomerular filtration rate measurement
CKD: Chronic kidney disease
PSM: Propensity score-matched analysis
CSS: Cancer-specific survival
MFS: Metastasis-free survival
PFS: Progression-free survival
CHD: Coronary heart disease
Hb: Hemoglobin
LDH: Lactic dehydrogenase
NSS: Nephron-sparing surgery
WIT: Warm ischemia time
ALP: Alkaline phosphatase
STE: Superselective transarterial embolization
