# Supplementary figures and images for: Laparoscopic partial versus radical nephrectomy for localized renal cell carcinoma over 4 cm
Source: J Cancer Res Clin Oncol. 2023 Nov 9;149(20):17837–48. doi: 10.1007/s00432-023-05487-3 (PMC10725398; doi:10.1007/s00432-023-05487-3)

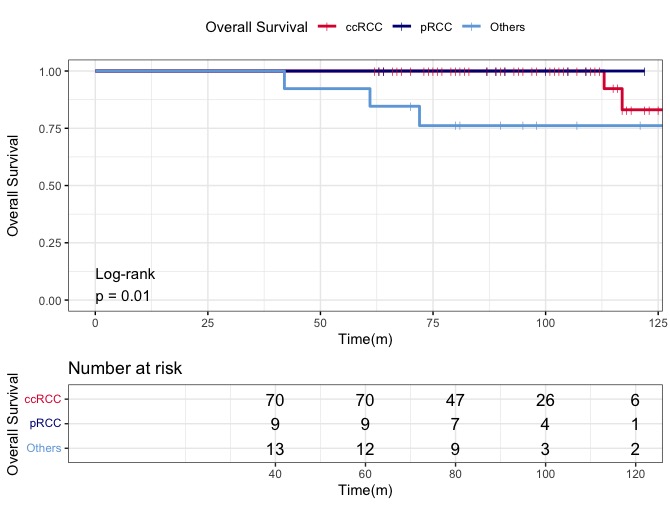

Supplement: Supplementary file 1 — Fig. S1: Kaplan–Meier curve of the influence of postoperative pathological types on OS. The red line represents clear cell renal cell carcinoma (ccRCC), the dark blue line represents papillary renal cell carcinoma (pRCC), and the light blue line represents other pathologic types. (TIFF 53 kb) [file 432_2023_5487_MOESM1_ESM.tiff]
