# Supplementary material for: Laparoscopic partial versus radical nephrectomy for localized renal cell carcinoma over 4 cm
Source: J Cancer Res Clin Oncol. 2023 Nov 9;149(20):17837–48. doi: 10.1007/s00432-023-05487-3 (PMC10725398; doi:10.1007/s00432-023-05487-3)
Supplement: Supplementary file 2 — (DOCX 67 kb) [file 432_2023_5487_MOESM2_ESM.docx]

**Online Supplementary Material**

This supplementary material has been provided by the authors to give readers additional information regarding their study.

**Laparoscopic partial versus radical nephrectomy for localized renal cell carcinoma over 4cm**

**Contents**

Sup.1 Data on the final pathology of the total 151 patients. 1

Sup.2 The details on the use of emission computed tomography. 13

Table S1. Comparison of postoperative complications between the two groups. 14

Table S2. Comparison of postoperative histology between the two groups. 16

Table S3. Comparison of the occurrence of CKD before and after operation between the two groups. 17

Table S4. Univariate analysis of prognostic risk factors for MFS. 18

Table S5. Univariate analysis of prognostic risk factors for PFS. 19

Table S6. Univariate analysis of prognostic risk factors for CSS. 21

# Sup.1 Data on the final pathology of the total 151 patients.

| **Patients Number** | **Group** | **Tumor location** | **Tumor size** | **Histological classification** | **Pathological details** | **Invasion (Yes/No)** |
| --- | --- | --- | --- | --- | --- | --- |
| 1 | LRN | Left | 4.2x3.5x2.4cm | ccRCC | Tumor boundary was clear, vascular recidivism (-), nerve recidivism (-), no cancer recidivism was found in para-mass renal tissue. | No |
| 2 | LRN | Left | 5.1x5x4.2cm | ccRCC | Cancer tissue boundary is clear, vascular recidivism (-), nerve recidivism (-). No cancer was found in the renal tissue adjacent to the mass | No |
| 3 | LRN | Right | 5x4x3cm | chRCC | Vascular (-), lymphatic (-), neurological (-) | No |
| 4 | LRN | Right | 6x5x3cm | ccRCC | Invasive growth, not yet penetrating renal envelope, vascular recidivism (-), lymphatic recidivism (-), nerve recidivism (-) | Yes |
| 5 | LRN | Right | 11x10.5x9cm | ccRCC | Renal capsule, vascular recidivism (-), nerve recidivism (-), no tumor invasion was found in renal tissue beside mass | Yes |
| 6 | LRN | Right | 7.5x4.7x3cm | chRCC | Vascular recidivism (-), nerve recidivism (-), the tumor did not invade the renal capsule, and there was no cancer invasion in the kidney tissue of 1.5cm near the mass | No |
| 7 | LRN | Right | 7x5x3cm | ccRCC | No cancer infiltration was found in vascular recidivism (-), nerve recidivism (-), renal pelvis and ureter | No |
| 8 | LRN | Left | 8x7x5.5cm | ccRCC | The tumor boundary was clear, the vascular recidivism (-), the nerve recidivism (-), the renal tissue beside the mass, the broken end of the ureter and the renal pelvis were not cancerous. Hilar lymph nodes showed no metastasis | No |
| 9 | LPN | Right | 5.5x5x2cm | ccRCC | Vascular (-), lymphatic (-), neurological (-) | No |
| 10 | LRN | Right | 4.5cm | ccRCC | Tumor has not penetrated the envelope, vascular recidivism (-), lymphatic recidivism (-), nerve recidivism (-) | No |
| 11 | LRN | Left | 5x4x3cm | ccRCC | Tumor has not penetrated renal envelope, vascular recidivism (-), lymphatic recidivism (-), nerve recidivism (-) | No |
| 12 | LRN | Right | 5x5x4cm | ccRCC | Tumor has not penetrated the envelope, vascular recidivism (-), nerve recidivism (-) | No |
| 13 | LRN | Left | 5x4.5x3cm | ccRCC | Vascular recidivism (-), lymphatic recidivism (-), nerve recidivism (-), focal lymphocyte infiltration in para-mass renal tissue | No |
| 14 | LRN | Right | 5.5x5.5x2.5cm | ccRCC | Vascular recidivism (-), lymphatic recidivism (-), nerve recidivism (-), renal hyperemia and edema near mass | No |
| 15 | LRN | Left | 7x6.5x3.2cm | ccRCC | The tumor boundary was clear, protruding into the perirenal adipose tissue, vascular recidivism (-), nerve recidivism (-), and no cancerous tissue was found in the renal tissue beside the mass | No |
| 16 | LRN | Left | 8x5.5x3cm | pRCC | Vascular recidivism (+), lymphatic recidivism (-), nerve recidivism (-), paracancer renal hyperemia with a small amount of lymphocyte infiltration | Yes |
| 17 | LRN | Right | 5.8x4x3.6cm | ccRCC | Invasive growth of tumor tissue, vascular recidivism (+), nerve recidivism (-), no tumor infiltration was observed in the surrounding renal tissue | Yes |
| 18 | LRN | Right | 7.5x5.5x5cm | CRCC | The tumor envelope was intact, vascular (-), lymphatic (-), nerve (-), and pericancerous renal tissue showed multiple renal cysts | No |
| 19 | LPN | Right | 6x4x3.5cm | ccRCC | The tumor was well delimited, and a small amount of surrounding renal tissue was not invaded by cancer | No |
| 20 | LRN | Left | 4.2x3x2.5cm | ccRCC | The tumor showed invasive growth, vascular recidivism (-), lymphatic recidivism (-), nerve recidivism (-). | Yes |
| 21 | LRN | Left | 13x10x9cm | ccRCC | Vascular recidivism (-), nerve recidivism (-), satellite cancer nodules can be seen near the cancer | Yes |
| 22 | LRN | Left | 6cm | ccRCC | Vascular recidivism (-), nerve recidivism (-), 1.5cm renal tissue structure adjacent to cancer existed | No |
| 23 | LRN | Left | 4.5x4x4cm | ccRCC | Vascular recidivism (-), lymphatic recidivism (-), nerve recidivism (-) | No |
| 24 | LRN | Left | 5x5cm | ccRCC | Invasive growth, not yet penetrating renal parenchyma, vascular recidivism (-), lymphatic recidivism (-), nerve recidivism (-) | Yes |
| 25 | LPN | Left | 6.5x5x4cm | ccRCC | Vascular recidivism (-), neurological recidivism (-) | No |
| 26 | LRN | Left | 4.5x3x3cm | ccRCC | Cancer tissue is limited to intracapsular, vascular (-), lymphatic (-), nerve (-) | No |
| 27 | LRN | Right | 4.5x4x4cm | ccRCC | Vascular recidivism (-), nerve recidivism (-), no cancer recidivism was observed in perirenal fat | No |
| 28 | LRN | Right | 4.8x3.5x3.5cm | ccRCC | Vascular recidivism (-), nerve recidivism (-), and renal pelvis of 1.5cm adjacent to the mass were not found with cancer tissue | No |
| 29 | LRN | Right | 4.5x4x3.8cm | ccRCC | Vascular recidivism (-), nerve recidivism (-), no tumor invasion of renal pelvis and kidney of 1.5cm near mass | No |
| 30 | LRN | Left | 6x6x6cm | ccRCC | Vascular recidivism (-), nerve recidivism (-), no cancer recidivism in other renal tissues | No |
| 31 | LPN | Left | 4.5x4x3.3cm | ccRCC | There was no tumor invasion in kidney tissue of 1.5cm beside the mass | No |
| 32 | LRN | Left | 11x9x6cm | ccRCC | The cancer tissue was located in the renal envelope, and no recidivists were observed in perirenal fat | No |
| 33 | LRN | Left | 4.2x3.5x2cm | ccRCC | The peripheral renal tissue showed focal chronic interstitial inflammation. The ureteral margin was negative | No |
| 34 | LPN | Right | 6x4.5x3.5cm | ccRCC | Vascular recidivism (-), neurological recidivism (-) | No |
| 35 | LRN | Right | 12x10x9cm | ccRCC | The cut margin of the ureter was negative. There were 2 hilar lymph nodes and no cancer | No |
| 36 | LPN | Right | 4.2x4x3cm | ccRCC | Vascular recidivism (-), neurological recidivism (-) | No |
| 37 | LPN | Left | 6x4x3cm | pRCC | Vascular recidivism (-), neurological recidivism (-) | No |
| 38 | LPN | Left | 8.5x7x6cm | ccRCC | Negative incisal margin | No |
| 39 | LPN | Right | 5x4x2.8cm | ccRCC | Vascular recidivism (-), neurological recidivism (-) | No |
| 40 | LPN | Left | 5.2×5×4.5cm | MCRN-LMP | The cut margin was negative | No |
| 41 | LRN | Right | 6.5x6cm | CRCC | Vascular recidivism (-), neurological recidivism (-) | No |
| 42 | LPN | Left | 5x4x4cm | ccRCC | Nerve recidivism (-), vascular recidivism (-), surgical margin negative | No |
| 43 | LRN | Right | 6x5x4cm | ccRCC | Invasive growth, vascular involvement (±). The ureteral margin was negative | Yes |
| 44 | LRN | Right | 5cmx4cmx3cm | CRCC | Vascular recidivism (-), neurological recidivism (-) | No |
| 45 | LPN | Left | 4.5x2.5x1.5cm | pRCC | Vascular recidivism (-), neurological recidivism (-) | No |
| 46 | LRN | Left | 4.5x3.8x3cm | ccRCC | Nerve recidivism (-), vascular recidivism (+), ureteral margin negative | Yes |
| 47 | LRN | Left | 5x4x3.5cm | ccRCC | Vascular recidivism (-), lymphatic recidivism (-), nerve recidivism (-) | No |
| 48 | LPN | Right | 7x5x3.5cm | ccRCC | Nerve recidivism (-), vascular recidivism (-), surgical margin negative | No |
| 49 | LRN | Right | 9.5x9.5x9.5cm | ccRCC | Clear boundary, vascular recidivism (-), nerve recidivism (-), no tumor invasion was found in renal tissue and renal pelvis mucosa | No |
| 50 | LRN | Right | 5.9x5.5x4.3cm | ccRCC | The boundary was clear, and no tumor recidivism was observed in other kidneys | No |
| 51 | LRN | Right | 6.5×7×7.5cm | MiT family tRCC | Clear boundary, vascular recidivism (-), nerve recidivism (-), no tumor invasion was found in renal tissue and renal pelvis mucosa | No |
| 52 | LPN | Left | 8.4x6.5x4.5cm | ccRCC | Clear boundary, vascular recidivism (-), nerve recidivism (-), no tumor invasion was found in renal tissue and renal pelvis mucosa | No |
| 53 | LRN | Right | 6.5x6.5x6cm | Others | The tumor protruded into the pelvic cavity, the incision margin of the ureter was negative, and no tumor plug was found in the renal portal vein | No |
| 54 | LRN | Left | 7.5x7cm | ccRCC | Clear boundary, vascular recidivism (-), nerve recidivism (-), no tumor invasion was found in renal tissue and renal pelvis mucosa | No |
| 55 | LRN | Right | 8x8cm | ccRCC | Clear boundary, vascular recidivism (-), nerve recidivism (-), no tumor invasion was found in renal tissue and renal pelvis mucosa | No |
| 56 | LPN | Right | 4.5x3x3cm | ccRCC | Clear boundary, vascular recidivism (-), nerve recidivism (-), no tumor invasion was found in renal tissue and renal pelvis mucosa | No |
| 57 | LRN | Left | 11x8x3cm | pRCC | There was no renal sinus involvement in the tumor. There was no tumor invasion of ipsilateral adrenal gland, and the self-examination was negative | No |
| 58 | LRN | Left | 4.8x4.5cm | ccRCC | Perirenal interstitial chronic inflammatory cell infiltration, renal hilum, perirenal tissue and adrenal gland no tumor involvement. Both ureter and blood vessel were negative. No metastasis was found in 1 hilar lymph node by self-examination | No |
| 59 | LPN | Left | 5x4x2.5cm | pRCC | Vascular recidivism (-), neurological recidivism (-). A small amount of renal tissue can be seen around the tumor with obvious hemorrhagic necrosis fibrosis | No |
| 60 | LPN | Right | 6.5x6x5.5cm | pRCC | The cut margin of the severed right kidney was negative. No obvious vascular recidivism was observed | No |
| 61 | LRN | Left | 6.5x5cm | ccRCC | Resection margin of ureter was negative; No metastatic cancer was found in lymph nodes (0/3) in the fibrous adipose tissue of the renal hilar | No |
| 62 | LRN | Right | 7.5cm | CRCC | The tumor was confined to the renal parenchyma and was negative on self-examination | No |
| 63 | LRN | Right | 5.5x3.7x2.9cm | ccRCC | Vascular recidivism (-), neurological recidivism (-), tumor boundaries are clear. There was no tumor invasion in kidney tissue 1.5cm near the mass, and no carcinoma in renal pelvis and renal sinus. The ureteral margin was negative. | No |
| 64 | LRN | Right | 5.5x4.5x3cm | ccRCC | Tumor boundary was clear, no tumor recidivism was observed in the rest renal parenchyma, no obvious vascular and nerve recidivism was observed. No tumor recidivism was observed in renal portal vein. No tumor was found at the incisal margin of the right ureter | No |
| 65 | LRN | Left | 4.5x4.2x3cm | ccRCC | No obvious vascular and nerve invasion was observed. Simple small cyst formation was observed in the renal parenchyma, and no carcinoma infiltration was observed in the calyces. No tumor thrombus was found in the renal hilar vessels. The incision margin of the ureter was negative, and no cancer metastasis was observed | No |
| 66 | LPN | Right | 5.5x4.5x4.5cm | ccRCC | Vascular recidivism (-), lymphatic recidivism (-), nerve recidivism (-) | No |
| 67 | LRN | Right | 5.5x5x3.5cm | ccRCC | Focal tumor necrosis was observed without perirenal fat or renal sinus involvement. The ureteral margin was negative. | No |
| 68 | LRN | Left | 11x10x10cm | CRCC | Vascular recidivism (+), partial tumor hemorrhagic necrosis, ureteral resection margin negative | No |
| 69 | LRN | Left | 5.2x4.5x4cm | ccRCC | Vascular recidivism (-), neurological recidivism (-). No carcinoma infiltration was observed in the renal pelvis. The "left" ureter was negative | No |
| 70 | LRN | Right | 4.5x4.5x3cm | ccRCC | Tumor boundaries were clear, and the incision margin of the ureter was negative | No |
| 71 | LPN | Left | 5x3x3cm | MCRN-LMP | Tumor boundary was clear, vascular recidivism (-), nerve recidivism (-), tumor bleeding necrosis and fibrosis were obvious | No |
| 72 | LPN | Right | 4.3x3.7x2.8cm | ccRCC | Tumor boundary was clear, vascular recidivism (-), nerve recidivism (-) | No |
| 73 | LRN | Right | 5.5x5x4.6cm | ccRCC | The ureteral cut was negative. No cancer recidivism was observed in renal medulla | No |
| 74 | LRN | Right | 4.3x3.3x3cm | ccRCC | The tumor was not observed in renal envelope spread, renal sinus infiltration, and intravascular cancer thrombus | No |
| 75 | LRN | Right | 6.5x5.5x3.5cm | ccRCC | Tumor boundary was clear, vascular recidivism (-), nerve recidivism (-) | No |
| 76 | LPN | Left | 5x3.5x2.2cm | pRCC | Vascular recidivism (-), nerve recidivism (-), little renal tissue was seen around, and the incisal margin was negative | No |
| 77 | LRN | Left | 8.5x8x7cm | CRCC | Vascular recidivism (-), lymphatic recidivism (-), nerve recidivism (-) | No |
| 78 | LPN | Left | 4.5x3.2x3cm | ccRCC | Vascular recidivism (-), lymphatic recidivism (-), nerve recidivism (-) | No |
| 79 | LRN | Left | 4.4x3.8cm | ccRCC | Vascular recidivism (-), lymphatic recidivism (-), nerve recidivism (-) | No |
| 80 | LPN | Left | 8x6x5.5cm | ccRCC | Vascular recidivism (-), nerve recidivism (-), resection margin of surgical severed end negative | No |
| 81 | LRN | Left | 7x7x3.5cm | CRCC | Vascular recidivism (-), neurological recidivism (-). Chronic inflammation of the pelvic mucosa. The hilar vessel and the incision margin of the ureter were negative | No |
| 82 | LPN | Left | 4.2×3.5×3.3cm | SDH-deficient RCC | Vascular recidivism (-), lymphatic recidivism (-), nerve recidivism (-) | No |
| 83 | LPN | Left | 5.5x5x4.5cm | pRCC | Vascular recidivism (-), lymphatic recidivism (-), nerve recidivism (-) | No |
| 84 | LPN | Left | 7.5x5.5x2.0cm | ccRCC | Vascular recidivism (-), lymphatic recidivism (-), nerve recidivism (-) | No |
| 85 | LRN | Right | 7.5x7x6.5cm | ccRCC | Vascular recidivism (-), neurological recidivism (-). The ureteral margin was negative | No |
| 86 | LRN | Left | 4.5x4.5x4.5cm | ccRCC | There was no clear vascular and nerve recidivism, chronic inflammation of peripheral renal interstitial tissue with glomerular fibrosis. The incision margin of ureter and vessel were negative | No |
| 87 | LRN | Left | 4.5x3.5x5cm | ccRCC | No obvious vascular and nerve invasion was observed, no carcinoma invasion was observed in renal pelvis, calyces and sinus, and no carcinoma invasion was observed in renal portal | No |
| 88 | LPN | Left | 5.0x4.7x4.0cm | ccRCC | Vascular recidivism (-), lymphatic recidivism (-), nerve recidivism (-) | No |
| 89 | LPN | Left | 5x3x2cm | ccRCC | Vascular recidivism (-), neurological recidivism (-) | No |
| 90 | LRN | Right | 7x7x6.5cm | ccRCC | No obvious vascular and nerve recidivism was observed. The cut margin of the ureter was negative | No |
| 91 | LRN | Left | 4.2x3x3cm | ccRCC | No perirenal and renal sinus fat invasion was found in the tumor. Intravascular cancer thrombus was negative (-), nerve invasion was negative (-), and proximal renal portal blood vessels and ureteral incisal margin were negative | No |
| 92 | LPN | Left | 5x5x4cm | ccRCC | Vascular recidivism (-), nerve recidivism (-), surgical specimens were negative | No |
| 93 | LPN | Left | 4.6x4x4cm | pRCC | The tumor is confined to the renal parenchyma. Incision margin of specimen was negative | No |
| 94 | LRN | Left | 12x9x8cm | chRCC | No definite vascular and nerve recidivism was observed | No |
| 95 | LRN | Left | 12x7x7cm | HLRCC | The incision margin of the left hilar vessel was negative. The incision margin of the left ureter is adjacent to the tumor. Left adrenal tissue, no tumor recidivism | No |
| 96 | LRN | Right | 6x5.5x5.5cm | ccRCC | No recurrence of renal sinus and perirenal adipose tissue was observed, and the ureteral resection margin was negative | No |
| 97 | LRN | Left | 11.0x9.0x5.0cm | ccRCC | The cut margin of the ureter was negative. No cancer metastasis in hilar lymph nodes | No |
| 98 | LPN | Right | 4.5x3.2x3.5cm | ccRCC | Vascular recidivism (-), lymphatic recidivism (-), nerve recidivism (-) | No |
| 99 | LRN | Left | 7x5.5x4cm | chRCC | Vascular recidivism (-), lymphatic recidivism (-), nerve recidivism (-) | No |
| 100 | LPN | Right | 4.5x3.5x3.5cm | ccRCC | Vascular recidivism (-), lymphatic recidivism (-), nerve recidivism (-) | No |
| 101 | LRN | Right | 9.5x8.5x6cm | ccRCC | The incision margin of the broken ureter was negative; No metastasis was found in right hilar lymph nodes | No |
| 102 | LPN | Right | 5x4x3.5cm | ccRCC | Vascular recidivism (-), lymphatic recidivism (-), nerve recidivism (-) | No |
| 103 | LPN | Right | 7x7x4cm | ccRCC | No definite vascular and nerve recidivism was observed, and the operative margin was negative | No |
| 104 | LPN | Left | 5x4x3.8cm | ccRCC | Vascular recidivism (-), lymphatic recidivism (-), nerve recidivism (-) | No |
| 105 | LRN | Right | 8.0x7.0x7.0cm | ccRCC | No obvious nerve and vascular recidivism were observed. The cut margin of the ureter was negative | No |
| 106 | LPN | Left | 4.5x4x2.5cm | chRCC | A small number of chronic inflammatory cells infiltrated the renal interstitial, and the incisal margin was negative | No |
| 107 | LRN | Left | 5.5x5x5cm | chRCC | Close to the capsule, no extrarenal invasion, no clear vasculature and nerve recidivism, chronic inflammation of the renal pelvis mucosa, and negative incision margin of the renal hilar vessel and ureter | No |
| 108 | LPN | Left | 5x4.5x4cm | Others | Vascular recidivism (-), lymphatic recidivism (-), nerve recidivism (-) | No |
| 109 | LPN | Left | 7x5x4cm | ccRCC | Vascular recidivism (-), lymphatic recidivism (-), nerve recidivism (-) | No |
| 110 | LRN | Left | 7.0x6.5x6.0cm | ccRCC | Vascular recidivism (-), lymphatic recidivism (-), nerve recidivism (-) | No |
| 111 | LPN | Right | 5.0x4.8x4.0cm | ccRCC | Vascular recidivism (-), lymphatic recidivism (-), nerve recidivism (-) | No |
| 112 | LRN | Right | 6.5x6.5x5cm | ccRCC | The tumor was located in the renal parenchyma, no clear vasculature or nerve recidivism was observed, and the incision margin of the ureteral and vascular severed ends were negative | No |
| 113 | LPN | Right | 5x3x4cm | ccRCC | Vascular recidivism (-), lymphatic recidivism (-), nerve recidivism (-) | No |
| 114 | LRN | Right | 5.2x4.6x5cm | ccRCC | Vascular recidivism (-), lymphatic recidivism (-), nerve recidivism (-) | No |
| 115 | LRN | Left | 5.8x5.6x2.5cm | ccRCC | The boundary between tumor and surrounding renal tissue was clear, nerve recidivism (-), vascular recidivism (-); The cut margin of the ureter was negative | No |
| 116 | LRN | Left | 7.5x6x4cm | ccRCC | The cancer tissue invaded the renal hilum, no clear vascular and nerve recidivists were observed, and some of the rest of the renal tissue had glomerular hyaline degeneration, renal tubule eosinophilic degeneration, and interstitial small vessels hyperplasia and congestion. The incisal margin of the broken end of the vessel was negative, the incisal margin of the broken end of the ureter was negative, and some ureteral mucosa had mild ordinary hyperplasia | Yes |
| 117 | LPN | Right | 7.0x6.8x6.5cm | ccRCC | Vascular recidivism (-), lymphatic recidivism (-), nerve recidivism (-) | No |
| 118 | LRN | Left | 5.5x5x4.5cm | ccRCC | The tumor is close to the renal pelvis adipose tissue. Chronic inflammation of the mucous membrane of the pyelouurinary tract. No tumor was found in ureteral incisal margin and hilar vessels by self-examination | No |
| 119 | LRN | Left | 6x4.5x3.5cm | ccRCC | Local hemorrhage and lymphocyte infiltration in tumor, vascular recidivism (-), nerve recidivism (-), resection margin of left ureter and renal portal vessel were negative. No tumor recidivism was observed in the left adrenal tissue. There was 1 paratumbral lymph node with no metastasis | No |
| 120 | LRN | Right | 6cm | ccRCC | No vascular or nerve recidivism was observed, and necrosis was observed within the tumor | No |
| 121 | LRN | Left | 5.5x4.2x4cm | ccRCC | No renal sinus and perirenal fat invasion were observed. No intravascular infiltration of renal hilar was observed. No renal pelvis involvement was observed. There was no tumor infiltration in ipsilateral adrenal tissue. | No |
| 122 | LPN | Right | 4.5x4x3.6cm | ccRCC | No definite vascular and nerve recidivism was found, and the incision margin of the surgical specimen was negative | No |
| 123 | LPN | Right | 6x5cm | ccRCC | Vascular recidivism (-), lymphatic recidivism (-), nerve recidivism (-) | No |
| 124 | LRN | Left | 6.2x6x4.3cm | ccRCC | Vascular recidivism (-), lymphatic recidivism (-), nerve recidivism (-) | No |
| 125 | LRN | Left | 5.5cm | ccRCC | No definite vasculature and nerve recidivism were observed, and the boundary with the surrounding renal tissue was clear | No |
| 126 | LRN | Left | 6x6x6cm | ccRCC | The tumor is located in the renal parenchyma and has not penetrated the envelope, blood vessels (-), nerves (-). Renal pelvis and ureter were negative. No tumor involvement was observed in adrenal tissue. | No |
| 127 | LRN | Right | 8x7x6cm | ccRCC | The mass was located in the renal tissue, no clear vasculature or nerve recidivism was observed, and the incision margin of the ureter was negative | No |
| 128 | LPN | Right | 4.5x4.0x3.8cm | ccRCC | No definite vascular and nerve recidivism was found, and the incision margin of the surgical specimen was negative | No |
| 129 | LRN | Left | 9.5x8x6cm | ccRCC | No renal sinus and perirenal fat invasion were observed. There was no renal hilar vascular infiltration, no renal pelvis involvement and no renal hilar vascular disjunction | No |
| 130 | LRN | Left | 8.5x6.2x6.3cm | ccRCC | Tumor recidivism Renal capsule, vascular recidivism (+), nerve recidivism (-). Eosinophilic change of renal tubules in adjacent renal tissue, thickening of some vascular walls and hyalinoid change. No cancer recidivism was observed in renal pelvis and sinus. The incision margin of renal vein and ureter were negative. No cancer metastasis was found in the "left hilar" lymph nodes | Yes |
| 131 | LRN | Right | 9x7x3cm | ccRCC | Vascular (-), nerve (-), renal pelvis no cancer involvement, the tumor has not penetrated the renal envelope. Ureteral dissections and vascular dissections were negative | No |
| 132 | LPN | Left | 4.5x4x3.5cm | ccRCC | Vascular recidivism (-), lymphatic recidivism (-), nerve recidivism (-) | No |
| 133 | LRN | Left | 5x3.5x3cm | ccRCC | Vascular recidivism (-), nerve recidivism (-), no carcinoma infiltration in paratumbral renal tissue, no carcinoma infiltration in renal pelvis; The cut margin of the ureter was negative | No |
| 134 | LPN | Left | 4.5x4x2.5cm | URCC | Vascular (-), nerve (-); Surgical margins were negative | No |
| 135 | LRN | Right | 11x9x7cm | ccRCC | The peripheral boundary of the mass was clear, and it adhered to the renal pelvis tissue. The ureteral end showed chronic inflammation of the mucosa, and the incisal margin was negative | No |
| 136 | LPN | Right | 5.5x4x3.5cm | ccRCC | No definite vascular and nerve recidivism was observed | No |
| 137 | LRN | Right | 4.2x4x3.5cm | ccRCC | Vascular recidivism (-), lymphatic recidivism (-), nerve recidivism (-) | No |
| 138 | LRN | Left | 5x5x4cm | ccRCC | Tumor invasion of renal parenchyma and renal pelvis, nerve recidivism (-), vascular recidivism (-). Resection margin of ureteral resection was negative. No cancer was found in adrenal tissue | No |
| 139 | LRN | Right | 6x6x6cm | ccRCC | Vascular recidivism (-), nerve recidivism (-), clear boundary with renal tissue, ureteral disjunction negative | No |
| 140 | LPN | Left | 6.8×5×3.8cm | ccRCC | Vascular recidivism (-), lymphatic recidivism (-), nerve recidivism (-) | No |
| 141 | LRN | Right | 6.5x6x6cm | ccRCC | The cancer tissue was located in the renal parenchyma, no recidivism was observed in the renal pelvis, and no definite vasculature and nerve recidivism were observed. The cut margin of the ureter was negative | No |
| 142 | LPN | Right | 5x3x2.5cm | ccRCC | Vascular recidivism (-), lymphatic recidivism (-), nerve recidivism (-) | No |
| 143 | LRN | Left | 5.5x4x4.5cm | ccRCC | No extrarenal spread of tumor was observed, and no vascular and nerve invasion was observed | No |
| 144 | LRN | Left | 4.5x3.5x3cm | ccRCC | The incision margin of ureteral and vascularized ends was negative | No |
| 145 | LPN | Right | 5.5x3.5x3cm | pRCC | Vascular recidivism (-), lymphatic recidivism (-), nerve recidivism (-) | No |
| 146 | LRN | Left | 8x7x7cm | chRCC | Vascular recidivism (-), nerve recidivism (-), no carcinoma invasion of renal pelvis mucosa; The incision margin of the severed end of the ureter was negative, the severed end of the renal portal vein was negative, and no obvious tumor thrombus was found | No |
| 147 | LRN | Right | 4.5x4x4cm | pRCC | Large bleeding and necrosis were observed in the tumor bed, and no exact nerve or vascular invasion was observed. There was no obvious lesion in the renal tissue beside the swelling. The incision margin of the cholecystectomy duct was negative | No |
| 148 | LPN | Left | 4.5x4x3.5cm | ccRCC | Vascular recidivism (-), lymphatic recidivism (-), nerve recidivism (-) | No |
| 149 | LPN | Right | 4.3x3.5x3.5cm | ccRCC | Vascular recidivism (-), lymphatic recidivism (-), nerve recidivism (-) | No |
| 150 | LPN | Left | 6.5x5.5x5cm | ccRCC | Vascular recidivism (-), lymphatic recidivism (-), nerve recidivism (-) | No |
| 151 | LPN | Right | 4.5x3.5x3cm | ccRCC | Vascular recidivism (-), nerve recidivism (-), surgical margin negative | No |

ccRCC: clear cell renal cell carcinoma; pRCC: papillary renal cell carcinoma; chRCC: chromophobe renal cell carcinoma; MCRN-LMP: multilocular cystic renal neoplasm of low malignant potential; MiT family tRCC: microphthalmia transcription factor family translocation renal cell carcinoma; SDH-deficient RCC: succinate dehydrogenase – deficient renal cell carcinoma; URCC: undifferentiated renal carcinoma; HLRCC: hereditary leiomyomatosis and renal cell carcinoma.

# Sup.2 The details on the use of emission computed tomography.

This study used SPECT/CT kidney dynamic imaging (Symbia T16, Siemens, Munich, Germany) with the following scanning parameters: energy peak: 140 keV; window width: 20%; matrix: 64 × 64; and zoom: 1.26×. Using pertechnetate (99mTcO4), freshly leached on the same day, we labeled the DTPA strictly according to the aseptic requirements to obtain 99mTc-DTPA with radiochemical purity > 95%. The activity dose was 111–185 MBq/kg, and the volume was injected 0.5–1 mL, using a 2-mL syringe and No. 7 needle.

The activity dose was calculated according to the patient's weight. The syringe with the drug was placed at the center of the probe for full needle measurement for 1 min. The patient was positioned on the scanner table to include the kidneys, and bladder in the probe field of vision. The bolus of the imaging drug was quickly injected into an elbow vein, and continuous double kidney dynamic acquisition was immediately performed. The dynamic scanning protocol included the acquisition of 60 s of blood perfusion images in 1 s/frame, followed by 20–30 min of renal function dynamic images in 30 s/frame. Afterward, the counting value at the injection point was measured for 1 minute. Thereafter, the empty syringe was measured again for 1 min to evaluate the counting value of the residual radiopharmaceutical in the needle(Li et al., 2022).

# Table S1. Comparison of postoperative complications between the two groups.

Postoperative complications occurred in 22 patients in the LPN group and 15 patients in the LRN group, including postoperative fever, anemia, hypoproteinemia, postoperative bleeding, vomiting, pleural effusion, pulmonary infection, hematuria, abdominal distension, celialgia, peritoneal effusion, acute kidney injury, heart failure, septic shock, and pulmonary atelectasis, respectively. According to the frequency of each symptom, anemia, postoperative fever, hypoproteinemia and postoperative bleeding were common in LPN group. Postoperative fever, hypoproteinemia, anemia, vomiting and abdominal distension were common in LRN group.

| **Postoperative complications, n (%)** | **LPN (n^^^ = 22)** | **LRN (n^^^ = 15)** | ***P* value** |
| --- | --- | --- | --- |
| Fever | 11 (21.6%) | 6 (11.8%) | 0.549 |
| Anaemia | 12 (23.6%) | 5 (9.8%) | 0.204 |
| Hypoproteinemia | 11 (21.6%) | 6 (11.8%) | 0.549 |
| Postoperative bleeding | 4 (7.8%) | 1 (1.9%) | 0.314 |
| Vomiting | 0 (0%) | 4 (7.8%) | 0.010* |
| Pleural effusion | 2 (3.9%) | 1 (1.9%) | 0.791 |
| Pulmonary infection | 1 (1.9%) | 1 (1.9%) | 0.779 |
| Hematuria | 2 (3.9%) | 0 (0%) | 0.230 |
| Abdominal distension | 0 (0%) | 2 (3.9%) | 0.078 |
| Celialgia | 0 (0%) | 1 (1.9%) | 0.220 |
| Peritoneal effusion | 1 (1.9%) | 0 (0%) | 0.403 |
| Acute kidney injury | 0 (0%) | 1 (1.9%) | 0.220 |
| Heart failure | 0 (0%) | 1 (1.9%) | 0.220 |
| Septic shock | 0 (0%) | 1 (1.9%) | 0.220 |
| Pulmonary atelectasis | 1 (1.9%) | 0 (0%) | 0.403 |

n^^^ represents the number of patients with postoperative complications in each group;

Each indicators were presented as n (%);

When multiple symptoms occur in the same patient, they are statistically counted separately for each symptom or event.

* P<0.05 was considered statistically significant.

# Table S2. Comparison of postoperative histology between the two groups.

| **Variable** | **LPN** | **LRN** | ***P* value** |
| --- | --- | --- | --- |
| Pathology, n (%) | n = 51 | n = 51 | 0.099 |
| ccRCC | 37 (72.6%) | 40 (78.5%) |  |
| pRCC | 7 (13.8%) | 2 (3.9%) |  |
| chRCC | 1 (1.9%) | 6 (11.8%) |  |
| MCRN-LMP | 2 (3.9%) | 0 (0%) |  |
| MiT family tRCC | 0 (0%) | 1 (1.9%) |  |
| SDH-deficient RCC | 1 (1.9%) | 0 (0%) |  |
| URCC | 3 (5.9%) | 2 (3.9%) |  |
| WHO/ISUP grading classification, n (%) | n=44 | n=42 | 0.843 |
| Ⅰ | 10 (22.7%) | 9 (21.4%) |  |
| Ⅱ | 31 (70.5%) | 28 (66.7%) |  |
| Ⅲ | 2 (4.5%) | 4 (9.5%) |  |
| Ⅳ | 1 (2.3%) | 1 (2.4%) |  |
| pTNM stage, n (%) | n=51 | n=51 | 0.741 |
| pT1bN0Mx | 47 (92.2%) | 45 (88.2%) |  |
| pT2aN0Mx | 4 (7.8%) | 6 (11.8%) |  |

ccRCC: clear cell renal cell carcinoma; pRCC: papillary renal cell carcinoma; chRCC: chromophobe renal cell carcinoma; MCRN-LMP: multilocular cystic renal neoplasm of low malignant potential; MiT family tRCC: microphthalmia transcription factor family translocation renal cell carcinoma; SDH-deficient RCC: succinate dehydrogenase – deficient renal cell carcinoma; URCC: undifferentiated renal carcinoma; WHO/ISUP: The World Health Organisation/International Society of Urological Pathology; pTNM stage: pathologic of the tumour, nodes, and metastasis stage.

# Table S3. Comparison of the occurrence of CKD before and after operation between the two groups.

| **Time** | **Total (N = 102)** | **LPN (n = 51)** | **LRN (n = 51)** | ***P* value** |
| --- | --- | --- | --- | --- |
| Preoperative | 23 (22.6%) | 12 (23.5%) | 11 (21.6%) | > 0.99 |
| Post-3 month | 51 (50.0%) | 16 (31.4%) | 35 (68.6%) | < 0.001* |
| Post-6 month | 45 (44.1%) | 13 (25.5%) | 32 (62.8%) | < 0.001* |
| Post-12 month | 40 (39.2%) | 10 (19.6%) | 30 (58.8%) | < 0.001* |

* P<0.05 was considered statistically significant.

Each indicators were presented as n (%);

# Table S4. Univariate analysis of prognostic risk factors for MFS.

When examining all patients, univariate analysis showed that drinking (HR, 5.476；95%CI, 1.105 – 27.139; p = 0.037) was the prognostic factors of MFS.

| **Variable** | **MFS** | |
| --- | --- | --- |
|  | **Univariate** | |
|  | HR (95%CI) | P value |
| Age (years) | 1.007 (0.949 – 1.068) | 0.820 |
| Gender (male vs. female) | 0.024 (0.000 – 25.620) | 0.295 |
| BMI | 1.007 (0.767– 1.322) | 0.962 |
| Surgical method (LRN vs. LPN) | 1.106 (0.223 – 5.482) | 0.902 |
| Smoking (No vs. Yes) | 2.756 (0.556 – 13.656) | 0.214 |
| Drinking (No vs. Yes) | 5.476 (1.105 – 27.139) | 0.037* |
| Hypertension (No vs. Yes) | 0.321 (0.038 – 2.750) | 0.300 |
| Diabetes (No vs. Yes) | 0.043 (0.000 – 9.885e+03) | 0.618 |
| CHD (No vs. Yes) | 0.048 (0.000 – 1.480e+09) | 0.805 |
| Laterality (Left vs. Right) | 2.039 (0.373 – 11.134) | 0.411 |
| Tumor size | 1.343 (0.703 – 2.567) | 0.372 |
| pT stage (pT1b vs pT2a) | 1.992 (0.233 – 17.058) | 0.529 |
| Hb | 1.056 (0.988 – 1.129) | 0.109 |
| Ca | 0.016 (0.000 – 21.336) | 0.259 |
| SCr | 1.014 (0.967 – 1.063) | 0.578 |
| LDH | 1.002 (0.983 – 1.021) | 0.862 |
| ECT-GFR | 0.999 (0.958 – 1.040) | 0.947 |
| Preoperative CKD (No vs. Yes) | 0.726 (0.085 – 6.213) | 0.770 |
| ASA score (1 vs .2 vs.3) | 1.683 (0.321 – 8.830) | 0.538 |
| Operation time | 1.001 (0.984 – 1.018) | 0.897 |
| Intraoperative bleeding | 0.999 (0.995 – 1.003) | 0.671 |
| Hospital stay | 1.009 (0.777 – 1.311) | 0.947 |
| Postoperative complication (No vs. Yes) | 1.857 (0.375 – 9.199) | 0.449 |
| Postoperative SCr | 0.998 (0.972 – 1.026) | 0.909 |
| Postoperative ECT-GFR | 0.988 (0.945 – 1.034) | 0.612 |
| Postoperative CKD (No vs. Yes) | 2.180 (0.399 – 11.904) | 0.368 |
| Postoperative histology# | 1.581 (0.634 – 3.941) | 0.325 |

MFS: metastasis-free survival; HR: hazard ratio; BMI: body Mass Inde; CHD: coronary heart disease; Before surgery, all patients with a history of CHD were well controlled and without surgical contraindications; Hb:hemoglobin; Ca: serum calcium; SCr: serum creatinine; LDH: lactic dehydrogenase; ECT-GFR: emission computed tomography for glomerular filtration rate measurement; CKD: chronic kidney disease; ASA: American Society of Anesthesiologists.

# Represents postoperative histology (clear cell renal cell carcinoma vs. papillary renal cell carcinoma vs. other types). * P<0.05 was considered statistically significant; 95%CI: 95% confidence interval.

# Table S5. Univariate analysis of prognostic risk factors for PFS.

When examining all patients, univariate analysis showed that ASA score (HR, 4.998; 95%CI, 1.575 – 15.792; p = 0.006) was the prognostic factors of PFS.

| **Variable** | **PFS** | |
| --- | --- | --- |
|  | **Univariate** | |
|  | HR (95%CI) | P value |
| Age (years) | 1.053 (0.998 – 1.111) | 0.059 |
| Gender (male vs. female) | 0.212 (0.027 – 1.682) | 0.142 |
| BMI | 0.981 (0.743 – 1.135) | 0.431 |
| Surgical method (LRN vs. LPN) | 2.033 (0.508 – 8.132) | 0.316 |
| Smoking (No vs. Yes) | 1.903 (0.537 – 6.749) | 0.319 |
| Drinking (No vs. Yes) | 2.173 (0.558 – 8.462) | 0.263 |
| Hypertension (No vs. Yes) | 0.629 (0.162 – 2.444) | 0.503 |
| Diabetes (No vs. Yes) | 0.043 (0.000 – 619.600) | 0.520 |
| CHD (No vs. Yes) | 6.021 (0.740 – 48.976) | 0.093 |
| Laterality (Left vs. Right) | 1.067 (0.308 – 3.693) | 0.918 |
| Tumor size | 1.433 (0.876 – 2.342) | 0.152 |
| pT stage (pT1b vs pT2a) | 0.944 (0.119 – 7.496) | 0.956 |
| Hb | 1.000 (0.957 – 1.045) | 0.994 |
| Ca | 0.015 (0.000 – 4.919) | 0.155 |
| SCr | 1.015 (0.980 – 1.052) | 0.396 |
| LDH | 1.009 (0.998 – 1.019) | 0.110 |
| ECT-GFR | 0.992 (0.963 – 1.021) | 0.579 |
| Preoperative CKD (No vs. Yes) | 1.383 (0.356 – 5.378) | 0.640 |
| ASA score (1 vs .2 vs.3) | 4.998 (1.575 – 15.792) | 0.006* |
| Operation time | 0.996 (0.981 – 1.012) | 0.621 |
| Intraoperative bleeding | 0.997 (0.992 – 1.003) | 0.328 |
| Hospital stay | 0.987 (0.790 – 1.232) | 0.906 |
| Postoperative complication (No vs. Yes) | 2.362 (0.653 – 8.545) | 0.190 |
| Postoperative SCr | 1.005 (0.986 – 1.024) | 0.633 |
| Postoperative ECT-GFR | 0.977 (0.943 – 1.013) | 0.206 |
| Postoperative CKD (No vs. Yes) | 2.236 (0.611 – 9.183) | 0.212 |
| Postoperative histology# | 1.858 (0.946 – 3.652) | 0.072 |

PFS: progression-free survival; HR: hazard ratio; BMI: body Mass Inde; CHD: coronary heart disease; Before surgery, all patients with a history of CHD were well controlled and without surgical contraindications; Hb:hemoglobin; Ca: serum calcium; SCr: serum creatinine; LDH: lactic dehydrogenase; ECT-GFR: emission computed tomography for glomerular filtration rate measurement; CKD: chronic kidney disease; ASA: American Society of Anesthesiologists.

# Represents postoperative histology (clear cell renal cell carcinoma vs. papillary renal cell carcinoma vs. other types). * P<0.05 was considered statistically significant; 95%CI: 95% confidence interval.

# Table S6. Univariate analysis of prognostic risk factors for CSS.

No variables were independently associated with CSS in univariate analysis.

| **Variable** | **CSS** | |
| --- | --- | --- |
|  | **Univariate** | |
|  | HR (95%CI) | P value |
| Age (years) | 1.155 (0.000 – 1.482) | 0.255 |
| Gender (male vs. female) | 0.025 (0.062 – 7.231e+05) | 0.674 |
| BMI | 1.510 (0.749 – 3.043) | 0.250 |
| Surgical method (LRN vs. LPN) | 71.454 (0.000 – 7.049e+08) | 0.603 |
| Smoking (No vs. Yes) | 0.030 (0.000 – 3.861e+06) | 0.713 |
| Drinking (No vs. Yes) | 0.038 (0.000 – 3.669e+08) | 0.780 |
| Hypertension (No vs. Yes) | 119.650 (0.000 – 2.937e+09) | 0.582 |
| Diabetes (No vs. Yes) | 0.043 (0.000 – 8.692e+11) | 0.841 |
| CHD (No vs. Yes) | 0.048 (0.000 – 5.354e+24) | 0.921 |
| Laterality (Left vs. Right) | 65.289 (0.000 – 6.281e+08) | 0.610 |
| Tumor size | 1.876 (0.409 – 8.598) | 0.418 |
| pT stage (pT1b vs pT2a) | 0.043 (0.000 – 1.649e+11) | 0.831 |
| Hb | 0.995 (0.871 – 1.136) | 0.938 |
| Ca | 0.000 (0.000 – 7.129) | 0.078 |
| SCr | 1.063 (0.954 – 1.185) | 0.267 |
| LDH | 1.013 (0.988 – 1.039) | 0.320 |
| ECT-GFR | 0.903 (0.778 – 1.048) | 0.180 |
| Preoperative CKD (No vs. Yes) | 772.032 (0.000 – 2.576e+15) | 0.651 |
| ASA score (1 vs .2 vs.3) | 1.683 (0.027 – 103.488) | 0.804 |
| Operation time | 1.000 (0.957 – 1.044) | 0.988 |
| Intraoperative bleeding | 1.001 (0.996 – 1.006) | 0.695 |
| Hospital stay | 0.327 (0.053 – 2.016) | 0.228 |
| Postoperative complication (No vs. Yes) | 0.025 (0.000 – 7.231e+05) | 0.674 |
| Postoperative SCr | 0.976 (0.898 – 1.062) | 0.577 |
| Postoperative ECT-GFR | 0.976 (0.870 – 1.094) | 0.671 |
| Postoperative CKD (No vs. Yes) | 68.245 (0.000 – 6.606e+08) | 0.607 |
| Postoperative histology# | 107.377 (0.000 – 2.680e+11) | 0.672 |
| Local recurrence (No vs. Yes) | 0.049 (0.000 – 2.520e+35) | 0.944 |
| Distant metastatic diseases (No vs. Yes) | 1.000 (0.000 – 2.801e+03) | 0.900 |

CSS: cancer-specific survival; HR: hazard ratio; BMI: body Mass Inde; CHD: coronary heart disease; Before surgery, all patients with a history of CHD were well controlled and without surgical contraindications; Hb:hemoglobin; Ca: serum calcium; SCr: serum creatinine; LDH: lactic dehydrogenase; ECT-GFR: emission computed tomography for glomerular filtration rate measurement; CKD: chronic kidney disease; ASA: American Society of Anesthesiologists.

# Represents postoperative histology (clear cell renal cell carcinoma vs. papillary renal cell carcinoma vs. other types). * P<0.05 was considered statistically significant; 95%CI: 95% confidence interval.

Li J, Huang L, Luo Y, Zhang K, Wang J, Feng J, Liu J (2022). Inferences of SPECT renal dynamic imaging injection quality based on lung and abdominal aorta imaging features. Ann Nucl Med, 36(8): 710-716.https://doi.org/10.1007/s12149-022-01750-8
